# Supplementary material for: A Matter of Degrees: Latitudinal Variation in the Transcriptional Response to High and Low Temperatures in an Estuarine Cnidarian
Source: bioRxiv. 2026 Apr 16:2026.04.14.718487. Preprint. [Version 1] doi: 10.64898/2026.04.14.718487 (PMC13104882; doi:10.64898/2026.04.14.718487)
Supplement: Supplement 1 [file media-1.pdf]

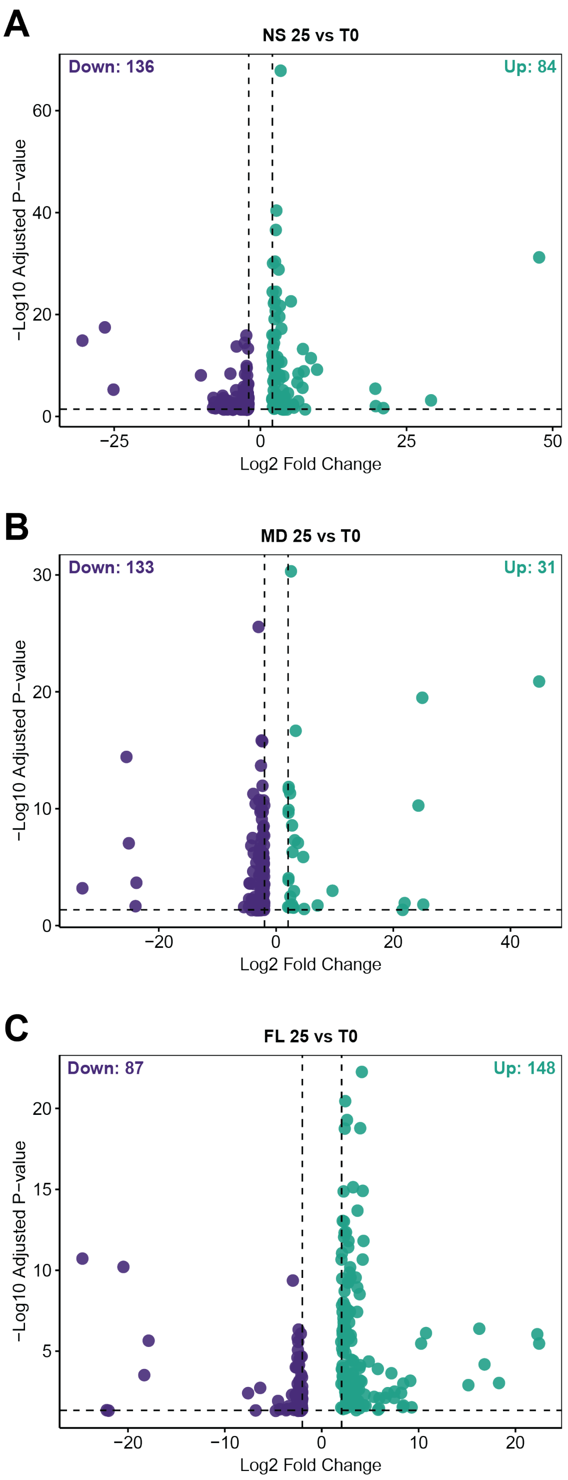

Figure S1. Volcano plots of differentially expressed genes in control samples (25 °C) relative to timepoint zero samples (T0). Significantly differentially downregulated genes (log2 fold change < -2, padj < 0.05) in purple (left) and upregulated genes (log2 fold change > 2, padj < 0.05) in teal (right). (**A**) Nova Scotia (NS). (**B**) Maryland (MD). (**C**) Florida (FL).

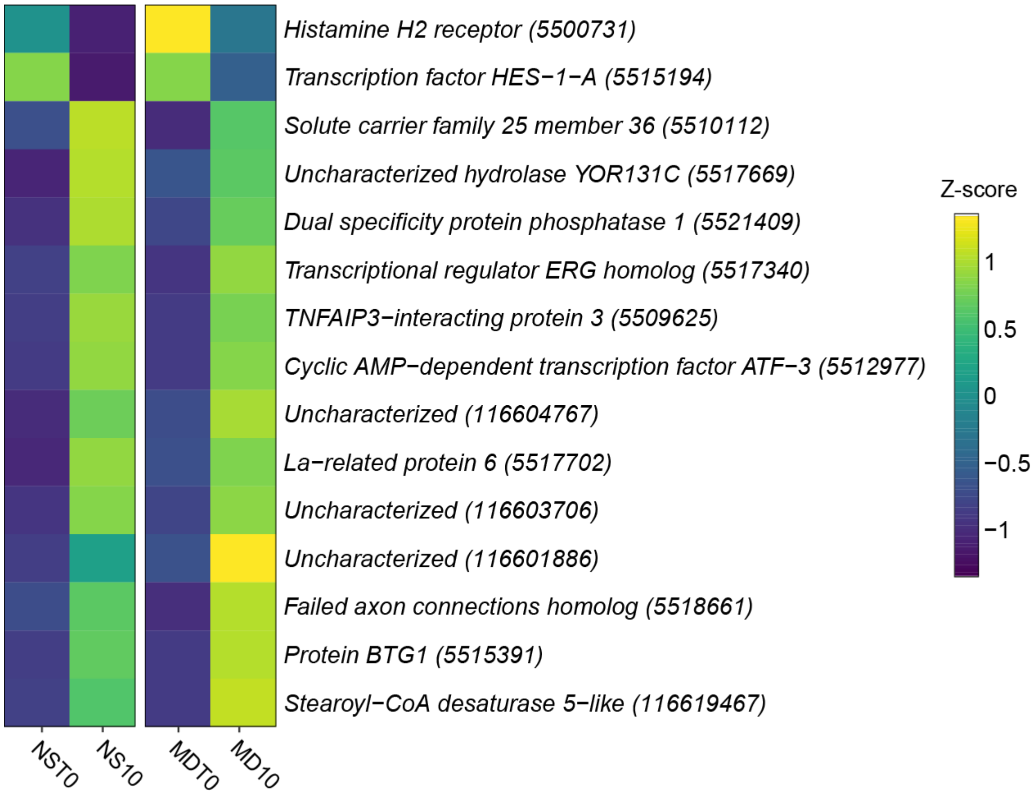

Figure S2. Heatmap visualizing z-scores of shared differentially expressed genes (DEGs) between Nova Scotia (NS) and Florida (FL) under cold stress (10 °C).

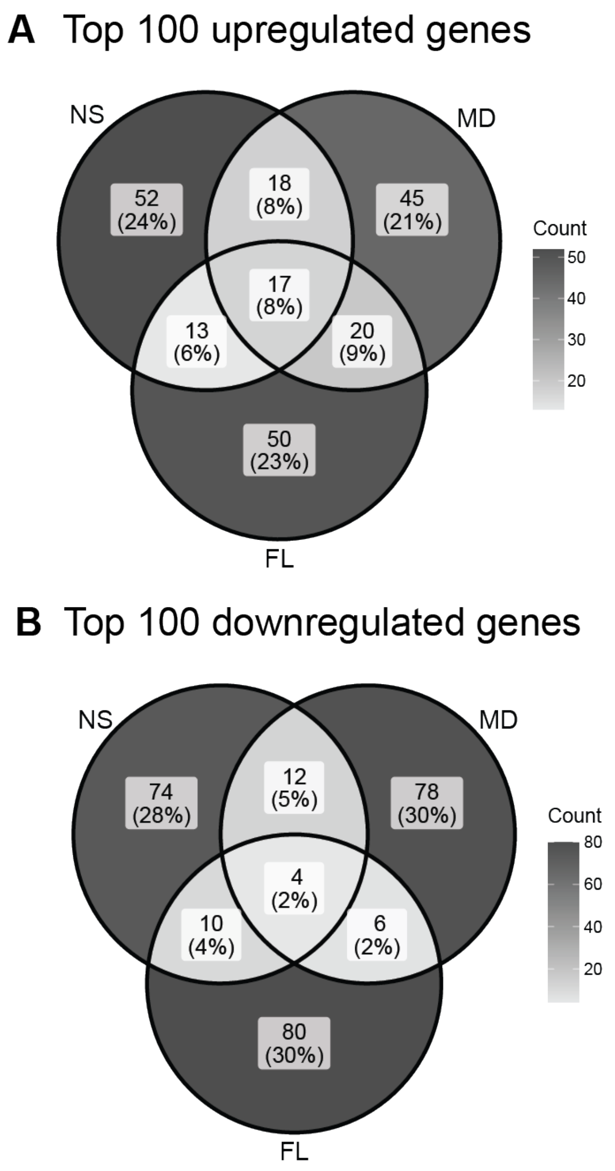

Figure S3. Venn diagrams of top 100 differentially expressed genes (DEGs) in heat stress (38 °C). (**A**) Top 100 upregulated DEGs with padj < 0.05. (**B**) Top 100 downregulated DEGs with padj < 0.05.

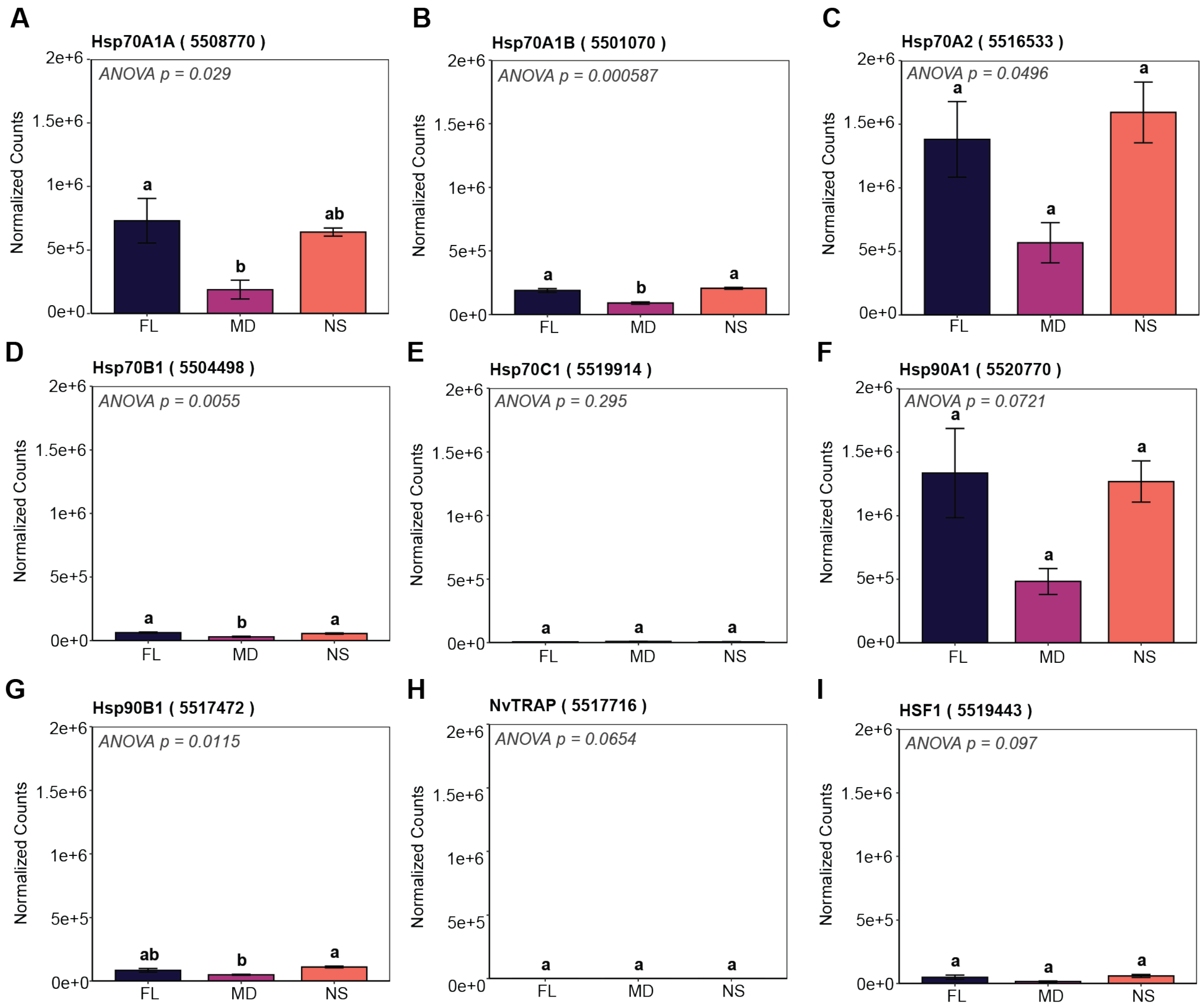

Figure S4. Heat shock response pathway gene expression at 38 °C across *Nematostella* populations. Gene expression visualized as the normalized read counts at 38 °C for all samples. One-way ANOVA conducted on normalized read counts. Letters on top of bars indicate statistical significance calculated with a post-hoc Tukey HSD test, where bars harboring unique letters represent statistically significant difference between means ($p < 0.05$). Error bars show the standard error of the mean. (**A**) Hsp70A1A expression. (**B**) Hsp70A1B expression. (**C**) Hsp70A2 expression. (**D**) Hsp70B1 expression. (**E**) Hsp70C1 expression. (**F**) Hsp90A1 expression. (**G**) Hsp90B1 expression. (**H**) NvTRAP expression. (**I**) HSF1 expression.

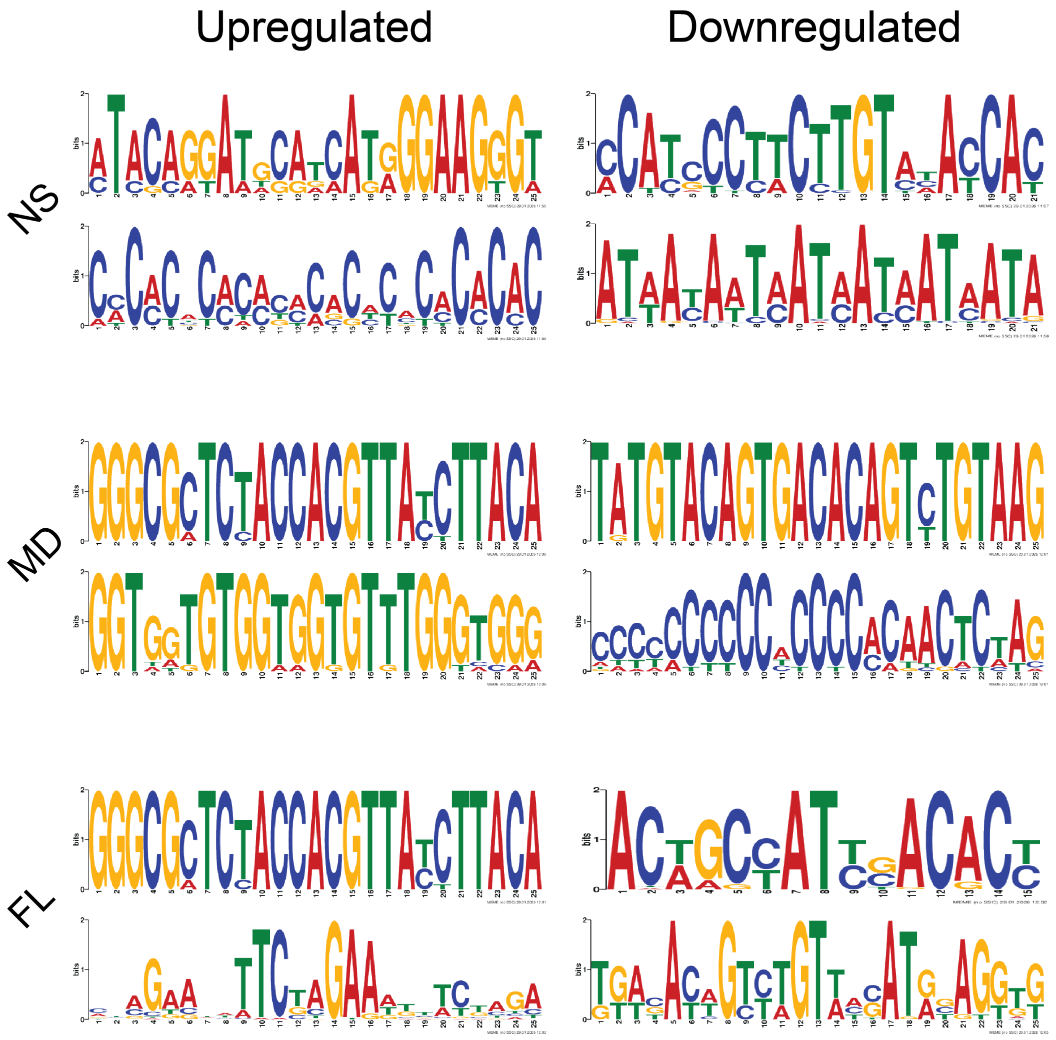

Figure S5. Motif enrichment analysis in differentially expressed genes under heat stress. Putative promoter sequences were used for the top 100 up- and downregulated genes from each population for enrichment analysis. Motifs are enriched motifs identified in each set of putative promoter sequences using the MEME program from MEME Suite (https://meme-suite.org/meme/).

Term	Count	Benjamini
**protein refolding**	**5**	**1.45E-09**
**response to heat**	**3**	**8.50E-05**
**cellular response to unfolded protein**	**2**	**9.16E-03**
vesicle-mediated transport	2	1.01E-01

Table S1. Gene ontology (GO) analysis of differentially expressed genes (DEGs) common in Maryland (MD) and Florida (FL) under cold stress (10 °C). Significantly enriched GO terms bolded.

Term	Count	Benjamini
**protein folding**	**8**	**0.03**
response to endoplasmic reticulum stress	4	0.11
regulation of transcription by RNA polymerase II	21	0.11
amino acid transmembrane transport	5	0.11
negative regulation of MAPK cascade	3	0.25
innate immune response	4	0.56
ubiquitin-dependent protein catabolic process	6	0.56
protein import into mitochondrial matrix	3	0.61
regulation of apoptotic process	5	0.71
positive regulation of proteasomal ubiquitin-dependent protein catabolic process	3	1.00
response to unfolded protein	2	1.00
protein phosphorylation	9	1.00
modification-dependent protein catabolic process	2	1.00

Table S2. Gene ontology (GO) analysis of differentially expressed genes (DEGs) common in Nova Scotia (NS), Maryland (MD), and Florida (FL) under heat stress (38 °C). Significantly enriched GO terms bolded.

Term	Count	Benjamini
**proteolysis**	**44**	**1.08E-16**
**chitin catabolic process**	**5**	**3.67E-04**
**lipid catabolic process**	**8**	**2.40E-03**
**carbohydrate metabolic process**	**11**	**2.53E-03**
**digestion**	**4**	**7.69E-03**
**ganglioside catabolic process**	**4**	**0.02**
negative regulation of membrane protein ectodomain proteolysis	3	0.10
collagen catabolic process	4	0.15
arachidonate secretion	4	0.18
phospholipid metabolic process	4	0.31
extracellular matrix organization	5	0.31
blood coagulation	2	0.48
sodium ion homeostasis	2	0.48
chemical synaptic transmission	8	0.61
positive regulation of fibroblast growth factor receptor signaling pathway	2	0.79
chloride transmembrane transport	4	0.79
potassium ion homeostasis	2	0.84
chloride ion homeostasis	2	0.84
mitochondrial transport	2	0.90
cell volume homeostasis	2	0.90

Table S3. Gene ontology (GO) analysis of upregulated genes unique to Nova Scotia (NS) under heat stress (38 °C). Significantly enriched GO terms bolded.

Term	Count	Benjamini
**DNA integration**	**37**	**6.59E-10**
**G protein-coupled receptor signaling pathway**	**66**	**6.10E-06**
double-strand break repair via homologous recombination	9	0.14
regulation of intracellular pH	6	0.37
DNA replication	9	0.52
monoatomic ion transmembrane transport	8	0.70
transcription preinitiation complex assembly	6	1.00
cellular response to light stimulus	9	1.00
phototransduction	9	1.00
DNA-templated transcription initiation	6	1.00
methylation	9	1.00
mitotic cell cycle	6	1.00
mitotic spindle assembly checkpoint signaling	3	1.00
nervous system process	7	1.00

Table S4. Gene ontology (GO) analysis of downregulated genes unique to Nova Scotia (NS) under heat stress (38 °C). Significantly enriched GO terms bolded.

Term	Count	Benjamini
**nucleosome assembly**	**4**	**0.01**
proteolysis	4	0.28
G protein-coupled receptor signaling pathway	5	0.28

Table S5. Gene ontology (GO) analysis of top upregulated genes unique to Florida (FL) under heat stress (38 °C). Significantly enriched GO terms bolded.

Term	Count	Benjamini
DNA integration	5	0.07
G protein-coupled receptor signaling pathway	6	0.70

Table S6. Gene ontology (GO) analysis of top downregulated genes unique to Florida (FL) under heat stress (38 °C).

Term	Count	Benjamini
**Protein processing in endoplasmic reticulum**	**3**	**0.05**

Table S7. Gene ontology (GO) analysis of top upregulated genes unique to Maryland (MD) under heat stress (38 °C). Significantly enriched GO terms bolded.

Term	Count	Benjamini
**transcription preinitiation complex assembly**	**4**	**0.01**
**DNA-templated transcription initiation**	**4**	**0.01**
**mismatch repair**	**3**	**0.03**

Table S8. Gene ontology (GO) analysis of top downregulated genes unique to Maryland (MD) under heat stress (38 °C). Significantly enriched GO terms bolded.
